# The Facile Construction of Defect-Engineered and Surface-Modified UiO-66 MOFs for Promising Oxidative Desulfurization Performance

**DOI:** 10.3390/nano15120931

**Published:** 2025-06-15

**Authors:** Chao Wang, Junchao Ding, Haoyu Wu, Jiaxuan Zhang, Jing Xu, Ying Zhang, Mindan Ma, Ming Zhang, Hongping Li

**Affiliations:** 1 School of the Environment and Safety Engineering, School of Emergency Management, Jiangsu University, Zhenjiang 212013, Chinaujswuhaoyu@126.com (H.W.);; 2 Institute for Energy Research, Jiangsu University, Zhenjiang 212013, China; zm@ujs.edu.cn

**Keywords:** UiO-66, surface modification, oxidative desulfurization, dibenzothiophene, hydrogen peroxide

## Abstract

The effective and deep removal of unreactive sulfides to achieve ultra-low-sulfur or sulfur-free oils has recently attracted extensive attention. In this work, a series of UiO-66 based catalysts have been prepared facilely for the effective removal of unreactive sulfides. Here, the incorporation of nitro functional groups into UiO-66, along with the construction of defects, results in remarkable sulfur removal for dibenzothiophene (DBT), achieving oil with sulfur content of less than 1 ppm. The successful construction of the designed catalysts was verified through a series of characterization studies. The exposed unsaturated metal sites help provide significantly more active reaction sites. In addition, the incorporated nitro group, with its electron-withdrawing property, would help increase the Lewis acidity of the catalytic metal sites. Thus, the catalytic oxidative capability of the designed UiO-66-based catalysts would be significantly increased. The enhanced catalytic oxidative performance helps ensure acceptable sulfur removal for oils with much higher sulfur concentrations. Additionally, the catalyst developed in this work can also be used to remove the derivatives of DBT with even lower reactivity. The relatively mild reaction conditions, combined with the exceptional sulfur removal, demonstrate the practicality of this reaction system.

## 1. Introduction

More recently, air pollution caused by exhaust emissions from billions of vehicles worldwide has become one of the most serious environmental issues [1,2,3]. Effective and deep desulfurization of refined oil products would help to notably reduce or eliminate this air pollution [4]. Although the hydrodesulfurization (HDS) technique applied in modern industrial processes could remove some sulfides (such as thiols, thioethers, etc.) effectively, other unreactive sulfides, like dibenzothiophene (DBT) and its derivatives, are still hard to remove, requiring harsh reaction conditions [5]. Thus, many other non-HDS strategies have been developed, including adsorptive desulfurization, bio-desulfurization, extractive desulfurization, and oxidative desulfurization (ODS) [6,7,8]. Around the numerous newly developed non-HDS strategies [9], the ODS procedure would be an efficient approach to eliminate unreactive aromatic sulfides in combination with traditional HDS procedures [10]. Through this process, an appropriate oxidant, such as hydrogen peroxide (H_2_O_2_) and aqueous or molecular oxygen (O_2_), was catalyzed to reactive oxygen species [11]. Subsequently, DBT and its derivatives are oxidized to their corresponding sulfones by the generated reactive oxygen species, which can then be naturally separated from the oil phase, resulting in oils with ultra-low sulfur concentrations [12]. Thus, exploring appropriate catalysts for ODS strategy is a critical issue that needs to be addressed.

Among the various candidates for the ODS procedure, metal–organic frameworks (MOFs)-based catalysts emerge as promising candidates for the catalytic oxidation of unreactive sulfides [13]. Here, the synthesized MOF materials are often used as ambient carriers due to their outstanding specific surface areas. Here, the active centers, including noble-metal nanoparticles [14], polyoxometalates [15], and inorganic nitrogen-based graphene-like materials [16], are loaded onto the surface of the MOF carriers or encapsulated in the hollow cavities of the MOF carriers. However, the leaching of active centers often occurs after several runs. In addition, the synthesis procedure of these catalysts often involves more than two steps. Thus, the construction of pure MOF catalysts with facile synthesis procedures merits in-depth investigation [17,18]. Their distinct metal sites and high specific surface areas provide the potential to function as reliable catalysts. In addition, the tunable defect sites and graftable surface functional groups can both contribute to elevating their catalytic capabilities [19,20].

UiO-66, as a Zr-based carboxylate MOF with a wide pH tolerance range, reliable thermal and mechanical stability, and superior ligand compatibility, is often selected for adsorption, chemical sensing, and catalytic reaction [21,22,23]. The highly stable structure of UiO-66 suggests that its fundamental framework would be retained after being modified through various strategies. Although the Zr-O clusters in the UiO-66 framework are fully coordinated theoretically, defects could still be constructed through a series of strategies [24]. The thermal activation of the synthesized MOFs with low boiling point solvents, such as methanol, is a commonly used strategy for producing defects [25]. Thereafter, catalytical metal sites would be exposed effectively, ensuring desirable catalytic oxidation activity. However, the thermal activation procedure is often carried out under high temperature or vacuum conditions, making the synthesis process more complex. In addition, it may lead to the release of potentially volatile organic pollutants. The incorporation of organic ligands with fewer coordination sites, along with commonly used organic ligands, is another strategy for producing MOFs with defects [26]. Here, no additional procedures are needed. However, the amount of different organic ligands added needs to be controlled precisely, or else the basic framework of the MOFs might collapse. Meanwhile, the substituents in organic ligands with different properties can influence the redox potential of metal sites, thereby further affecting their catalytic oxidation ability. Specifically, the substituent with electron-withdrawing features would help to increase the Lewis acidity of the catalytical metal sites, enhancing its catalytic activity consequently [27].

UiO-66-based catalysts, owing to their porous structure and high surface area, have drawn significant attention for applications in ODS. However, pristine UiO-66 exhibits limited oxidative catalytic activity. Therefore, various strategies have been developed to achieve improved ODS performance. One effective approach is the incorporation of additional catalytic metal sites into the UiO-66 framework. This can be accomplished through two main methods. The first and most commonly used method involves co-introducing metal salts with ZrCl_4_ during synthesis to directly obtain metal-doped UiO-66. Using this strategy, Ce [28], W [29], Hf [30], and Ti-doped [31] UiO-66 catalysts have been successfully synthesized, all showing promising ODS performance. The second method involves a post-synthetic modification: pristine UiO-66 is first synthesized and then immersed in a metal salt solution to introduce the desired metal species. Using this method, V [32] and Ti-doped [33,34,35] UiO-66 catalysts with acceptable ODS activity have been reported. Another commonly used strategy is to introduce catalytic polyoxometalates (POMs) and encapsulate them within the porous structure of UiO-66 [36,37,38,39,40], which also demonstrates effective sulfide removal. Moreover, loading ionic liquids [41] or metal oxides [42] onto the surface of UiO-66 can also enhance the catalytic performance of the active sites, benefiting from the well-developed porous structure and high specific surface area of the UiO-66 support. However, the above studies primarily focus on the introduction of catalytic sites, while the liberation of the catalytic potential of the inherent Lewis acidic Zr sites has received little attention.

Herein, UiO-66 with unsaturated metal sites (marked as D-UiO-66) was prepared by adding a specific amount of benzoic acid (BZA) together with terephthalic acid. Meanwhile, UiO-66 with -NO_2_ group (marked as UiO-66-NO_2_) was fabricated by utilizing 2-nitro terephthalic acid in place of terephthalic acid. At last, UiO-66 with both unsaturated metal sites and -NO_2_ group (marked as D-UiO-66-NO_2_) was constructed by employing both BZA and 2-nitro terephthalic acid as the organic ligand. Then, the successful construction of D-UiO-66, UiO-66-NO_2_, and D-UiO-66-NO_2_ was verified through a series of characterization strategies. The desulfurization capability of the synthesized UiO-66-based catalysts was measured through the effective oxidation of DBT and its derivatives to their corresponding sulfones, realizing the deep removal of unreactive sulfides from the oil phase. Under the optimized reaction conditions, sulfur removal of 99.8% (<1 ppm) could be obtained after the reaction for 100 min under relatively mild reaction conditions. Also, desirable sulfur removal could be achieved for oils with different substrates and sulfur concentrations. The exceptional sulfur removal capability of the UiO-66-based catalyst, featuring unsaturated metal sites and surface-incorporated -NO_2_ groups, indicates the construction of similar MOF-based catalysts with comparable strategies for promising catalytic performance.

## 2. Materials and Methods

The details about the characterization methods and the detection of sulfur concentration are listed in the Supporting Information.

### 2.1. Reagents

n-Dodecane (97%), 2-nitro terephthalic acid (98%), and benzoic acid (99%) were purchased from Shanghai Macklin Biochemical Technology Co., Ltd. (Shanghai, China) n-Hexadecane (98%), N, N-dimethylformamide (DMF, AR), dibenzothiophene (98%), 4-methyl-dibenzothiophene (98%), and 4,6-dimethyl-dibenzothiophene (97%) were purchased from Shanghai Aladdin Biochemical Technology Co., Ltd. (Shanghai, China) Zirconium chloride (99.9%) was obtained from Alfa Aesar. Terephthalic acid (99%) was obtained from Beijing Vokai Biotechnology Co., Ltd. (Beijing, China) Acetonitrile (AR), 30% H_2_O_2_ (99.5%), and anhydrous ethanol (AR) were purchased from Sinopharm Chemical Reagent Co., Ltd. (Shanghai, China).

### 2.2. Synthesis of UiO-66

Here, UiO-66 was synthesized through a typical solvothermal synthesis procedure. A dual molar amount of ZrCl_4_ (3 mmol, 700 mg) and terephthalic acid (3 mmol, 498 mg) was added to the polytetrafluoroethylene-lined autoclave. Then, 60 mL of DMF was added, and the mixture was continuously stirred for 30 min. After that, the autoclave was placed in an oven and reacted for 2 h under 130 °C. After the autoclave cooled naturally, the product was separated through centrifugation and washed three times with hot ethanol. At last, the obtained product was dried in a vacuum drying oven for 12 h to yield a white powder, which is named UiO-66.

### 2.3. Synthesis of UiO-66-Based Catalysts

The synthesis of UiO-66 with unsaturated metal sites is similar to that of pristine UiO-66, except for the initial incorporation of an additional 60 mmol of BZA alongside terephthalic acid. The final obtained product was named D-UiO-66. For the synthesis procedure of UiO-66 with -NO_2_ groups, 3 mmol (633 mg) of 2-nitro terephthalic acid was added instead of terephthalic acid. The final obtained powder was named UiO-66-NO_2_. Finally, for the synthesis of UiO-66 with both unsaturated metal sites and -NO_2_ groups, 3 mmol (633 mg) of 2-nitro terephthalic acid was used in place of terephthalic acid, along with an additional 60 mmol of BZA. The resulting product was designated as D-UiO-66-NO_2_.

### 2.4. Desulfurization Procedure

The desulfurization experiment was conducted in a homemade jacketed three-necked flask. Here, the reaction temperature was controlled by a super constant temperature water bath. The model oil was prepared by dissolving a specific amount of DBT or its derivatives in dodecane. The initial sulfur concentration for oils containing DBT, 4-MDBT, or 4,6-DMDBT was set at 200 ppm. Additionally, oils with sulfur concentrations of 500 ppm and 1000 ppm were prepared using DBT as the substrate. In a typical reaction procedure, 75 mg of the prepared catalyst, 5 mL of acetonitrile (MeCN), and 5 mL of model oil were sequentially added into the flask. The reaction temperature was set to 60 °C, and the system was stirred with a magnetic stirrer for 20 min initially. Then, a certain amount of H_2_O_2_ was injected into the reaction system to initiate the oxidative desulfurization procedure. The oil phase was taken every 20 min to determine its sulfur concentration.

The desulfurization of the oil was calculated by Formula (1).

(1) $$sulfur\;removal=\frac{C_{0}-C_{t}}{C_{0}}\times 100\%$$

Here, C_0_ and C*_t_* indicate the sulfur concentration at 0 min and t min, respectively.

## 3. Results and Discussion

### 3.1. The Characterization of the Synthesized UiO-66-Based Catalysts

**Figure 1:**
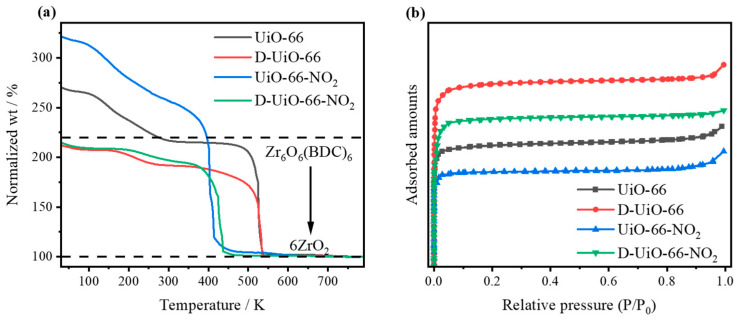
(**a**) TGA curves (relative to ZrO_2_) and (**b**) N_2_ adsorption–desorption isotherms.

All the samples prepared were initially characterized by thermogravimetric analysis (TGA, Figure 1a). The final weight of each tested sample was normalized to 100%, assuming ZrO_2_ as the sole solid residue for all UiO-66-based samples at temperatures above 700 °C [43]. For the TGA curve of pristine UiO-66, the initial weight loss below 150 °C can be attributed to the evaporation of trapped volatile solvents [43]. The second stage, occurring between 150 °C and 350 °C, is primarily due to the dehydroxylation of the Zr_6_ cluster nodes and the removal of residual monocarboxylate ligands [44]. The final weight loss above 350 °C corresponds to the decomposition of the MOF [45]. For each mole of dehydroxylated ideal UiO-66 (Zr_6_O_6_(BDC)_6_), six moles of ZrO_2_ are ultimately formed. Therefore, the theoretical mass of Zr_6_O_6_(BDC)_6_ is 220.2% relative to the final ZrO_2_ residue. However, the actual value is slightly lower than the theoretical one, indicating a small extent of missing linkers during the synthesis process. For D-UiO-66 and D-UiO-66-NO_2_, the significant reduction in the second plateau values suggests an increased degree of linker missing induced by BZA addition during synthesis, resulting in abundant missing-linker defects. In addition, for UiO-66 constructed using nitro group substituted linkers, a decreased decomposition temperature was observed [46,47]. The relative mass of UiO-66-NO_2_ at 350 °C exceeds 220.2%, which can be attributed to the presence of the nitro group in the linker.

The structures of the synthesized UiO-66-based samples were further characterized by N_2_ adsorption–desorption measurements (Figure 1b, Table S1). All samples exhibit typical type I isotherms, consistent with the microporous nature of the UiO-66 framework. However, the calculated Brunauer–Emmett–Teller (BET) specific surface areas of D-UiO-66 and D-UiO-66-NO_2_ are significantly higher than that of pristine UiO-66, which can be attributed to the presence of missing-linker defects [48,49]. However, the specific surface areas of the samples containing nitro groups are significantly lower than those without, which could be attributed to the introduction of a bulky and heavy functional group [46].

**Figure 2:**
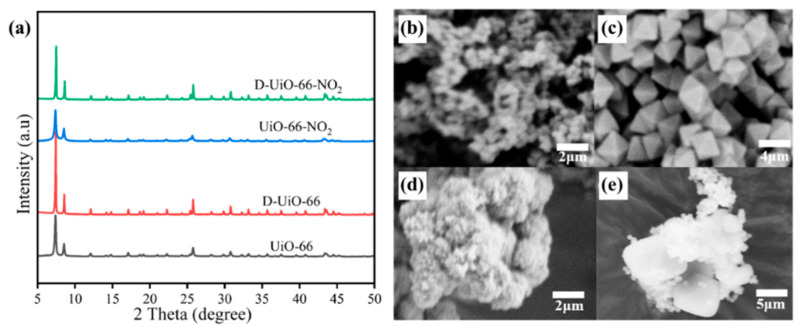
(**a**) XRD patterns of different UiO-66 based catalysts; SEM images of (**b**) UiO-66, (**c**) D-UiO-66, (**d**) UiO-66-NO_2_, and (**e**) D-UiO-66-NO_2_.

Then, the four kinds of catalysts produced were characterized by X-ray diffraction measurement (Figure 2a). All the synthesized catalysts show two distinct groups of peaks, with a strong diffraction peak at 7.4° attributed to the (111) facet, and a relatively weaker peak at 8.5° corresponding to the (200) facet [50]. Moreover, other characteristic peaks of UiO-66 could also be identified, including peaks appearing around 17.1°, 22.3°, 25.7°, 30.8°, 33.2°, and 43.4° [50]. However, after introducing the -NO_2_ group, no significant differences could be observed, implying that the incorporated functional group will not bring about any imperfections in its crystal [51]. Notably, the fabricated catalysts with defect sites show relatively superior crystallinity. This may be attributed to the in situ formation of metal cluster building blocks through the interaction between zirconium cations and BZA, which facilitates more controlled nucleation and consequently enhances crystallinity [52,53,54]. Moreover, it is worth noting that the increased crystallinity for catalysts with defect sites could also be observed from the scanning electron microscope (SEM) measurements (Figure 2b–e).

**Figure 3:**
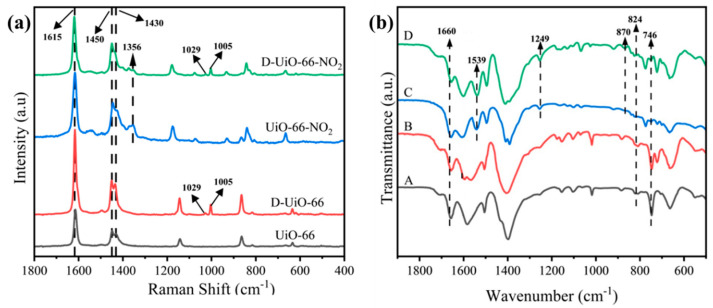
(**a**) Raman and (**b**) FT-IR analyses of UiO-66 based catalysts. A: UiO-66, B: D-UiO-66, C: UiO-66-NO_2_, and D: D-UiO-66-NO_2_.

To better understand the chemical structure of the fabricated catalysts, Raman spectra of all the samples were collected (Figure 3a). Here, all the catalysts show clearly characteristic peaks around 1615 cm^−1^, which would be assigned to the specific peak from the benzene ring of organic ligand [55,56]. The peaks observed around 1450 and 1430 cm^−1^ might be associated with the carboxyl groups in the organic ligand (O-C=O bond in carboxylate and C-C aromatic to carboxylate stretch) [56,57]. For D-UiO-66 and D-UiO-66-NO_2_, distinctive peaks for BZA could be observed at about 1029 and 1005 cm^−1^ [58]. And for UiO-66-NO_2_ and D-UiO-66-NO_2_, symmetrical stretch vibration of the -NO_2_ group surrounding 1356 cm^−1^ could also be identified [46]. The above inspections help to confirm the successful introduction of the organic ligand that we expect.

To gain a deeper awareness of the catalysts, Fourier transform infrared (FT-IR) spectra of the samples were collected simultaneously (Figure 3b). Here, for all the samples, the characteristic peaks of Zr-O could be found around 1156, 1100, and 1018 cm^−1^, illustrating effective coordination between the metal center and organic ligand [59,60]. Moreover, Zr-(OC) asymmetric stretching and *μ*_3_-O stretching could be discovered at 550 and 665 cm^−1^, respectively, indicating the construction of UiO-66 based frameworks simultaneously [60,61,62]. In addition, the peaks presented around 1660, 824, and 746 cm^−1^ for all the samples could be assigned to C=O stretching, C-H vibration, and C=C stretching from the organic ligand, respectively [60,63]. Notably, additional characteristic peaks could be detected around 1539, 1249, and 870 cm^−1^ for UiO-66-NO_2_ and D-UiO-66-NO_2_. The former peak and the latter two peaks would be associated with the asymmetric stretching of the -NO_2_ group and the stretching of C-N, respectively [64,65]. The above results indicate the successful maintenance of the UiO-66 framework in all the prepared samples. Moreover, the efficient incorporation of defects and functional groups was verified simultaneously, benefiting the enhancement of the oxidative catalytic capabilities.

### 3.2. Sulfur Removal with Different Reaction Systems

**Figure 4:**
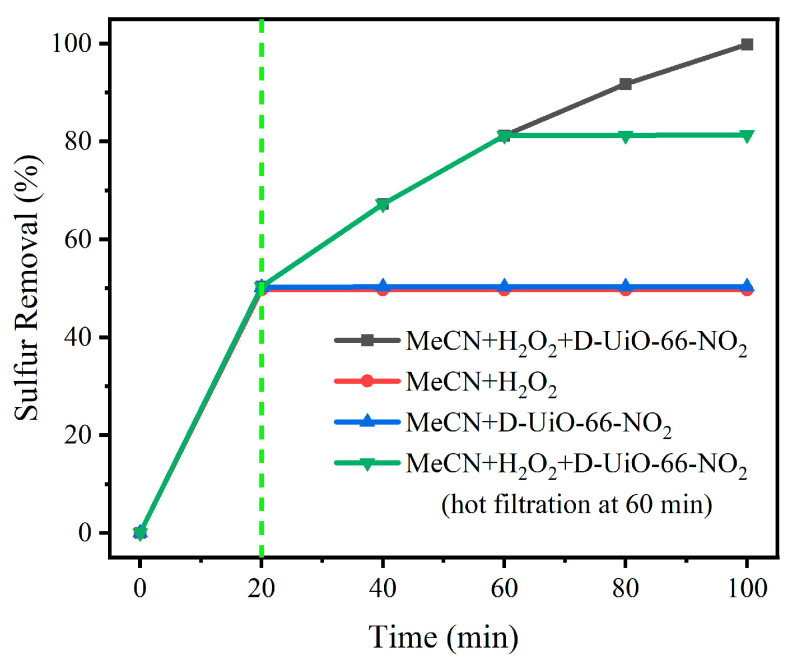
Sulfur removal with different reaction systems.

First of all, sulfur removal with different reaction systems was investigated and is shown in Figure 4. Here, DBT, as a typical stubborn sulfide in oils, was selected as the substrate. For each reaction adding H_2_O_2_, the oxidant was introduced following a 20 min extraction procedure with MeCN [66]. Here, for the reaction system without using any catalyst, no obvious sulfur removal could be viewed after extraction for 20 min, indicating that H_2_O_2_ would not be activated to reactive oxygen species with MeCN. In the absence of H_2_O_2_, no apparent increase in sulfur removal was observed after 20 min of extraction, illustrating that oxidative desulfurization could not occur in the absence of an oxidant. Thus, the catalyst and the added oxidant both display indispensable roles during the oxidative desulfurization procedure. Afterwards, sulfur removal of 99.8% could be noticed after another reaction for 80 min with adding both D-UiO-66-NO_2_ and H_2_O_2_. To verify the essential role of D-UiO-66-NO_2_ in a further step, hot filtration of the catalyst was conducted. Here, no evident increase in sulfur removal was observed after the hot filtration procedure. Thus, we might conclude that the added MeCN, H_2_O_2_, and D-UiO-66-NO₂ serve as the extractant, oxidant, and catalyst, respectively.

### 3.3. Sulfur Removal with Different UiO-66 Catalysts

**Figure 5:**
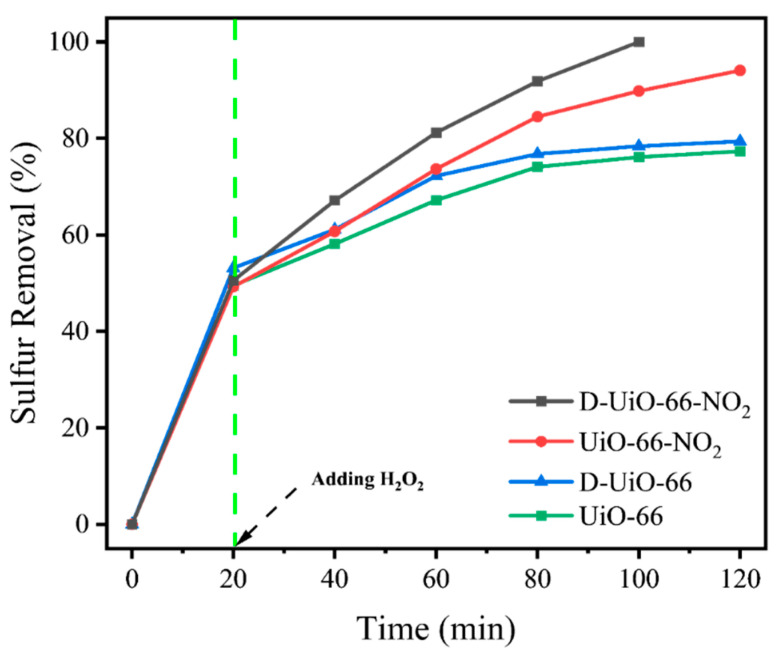
Sulfur removal for different UiO-66 based catalysts.

The UiO-66 catalysts gained with different features were utilized for the desulfurization of DBT, as a typical unreactive sulfide in oils (Figure 5). The sulfur removal observed during the initial 20 min can be mainly attributed to the extractive desulfurization capability of acetonitrile [66]. After adding H_2_O_2_, the sulfur removal for DBT increases continuously. For D-UiO-66-NO_2_, deep desulfurization (99.8%) could be obtained after reaction for another 80 min, obtaining oil with a sulfur concentration of less than 1 ppm. For UiO-66, sulfur removal of only 76.1% was received after another 100 min reaction, which indicates the indispensable role played by the constructed defect sites and incorporated -NO_2_ functional group. Compared to UiO-66 (76.1%), the increased sulfur removal for D-UiO-66 (78.4%) and UiO-66-NO_2_ (89.8%) indicates that the exposed uncoordinated metal sites and the introduction of the electron-withdrawing group both contribute to enhancing the oxidative catalytic capability. Compared with previously reported studies, the catalysts prepared in this work exhibit more moderate reaction conditions while achieving higher sulfur removal (Table 1).

For the unmodified UiO-66, a small number of inherent defects still exist, as confirmed by TGA measurements. In this case, the metal sites with Lewis acidity can catalyze H_2_O_2_ to generate reactive oxygen species, which subsequently oxidize DBT into its corresponding sulfone. However, due to the limited number of defects and the unenhanced Lewis acidity of the metal sites, deep desulfurization cannot be achieved. However, for UiO-66 with defects and functional groups, increased defect density and enhanced Lewis acidity can be observed. Due to the electron-withdrawing nature of the nitro group, the electron density at the Zr sites in UiO-66 is reduced through conjugation with the benzene ring [67]. As a result, the Zr sites exhibit an enhanced ability to accept electrons, thereby increasing their Lewis acidity and improving their acid-catalytic performance. The formation of Zr–OH and Zr–OH_2_ structures further facilitates the interaction between the hydrophilic oxidant [67,68], H_2_O_2_, and the Zr sites, promoting its catalytic conversion into peroxyl radicals. These reactive species then oxidize DBT into its corresponding sulfone, which can be readily separated from the oil phase due to its increased polarity, thus achieving deep desulfurization (Schemes S1 and S2).

**Table 1:** Catalytic ODS sulfur compounds over UiO-66-based catalysts by using H_2_O_2_.

Catalyst	Temp.	Time	O/S Ratio	DBT	4,6-DMDBT	Ref.
D-UiO-66-NO_2_	60 °C	100 min	7/1	99.8%	93.7%	This work
UiO-66	60 °C	150 min	12/1	100%	72.9%	[69]
UiO-66-NO_2_	60 °C	120 min	12/1	97%	—	[70]
UiO-66-free	60 °C	120 min	6/1	99.6%	—	[71]
UiO-66-MW	50 °C	180 min	13/1	95%	—	[72]

### 3.4. The Optimization of Oxidative Desulfurization Parameters

Afterward, D-UiO-66-NO_2_ was selected as a superior catalyst for extended investigations. The reaction temperature, oxidant dosage, and catalyst amount would influence the choice of reaction equipment and the overall cost of the desulfurization process. Thus, the optimization of reaction temperature, oxidant dosage, and catalyst amount was conducted through a series of experiments.

**Figure 6:**
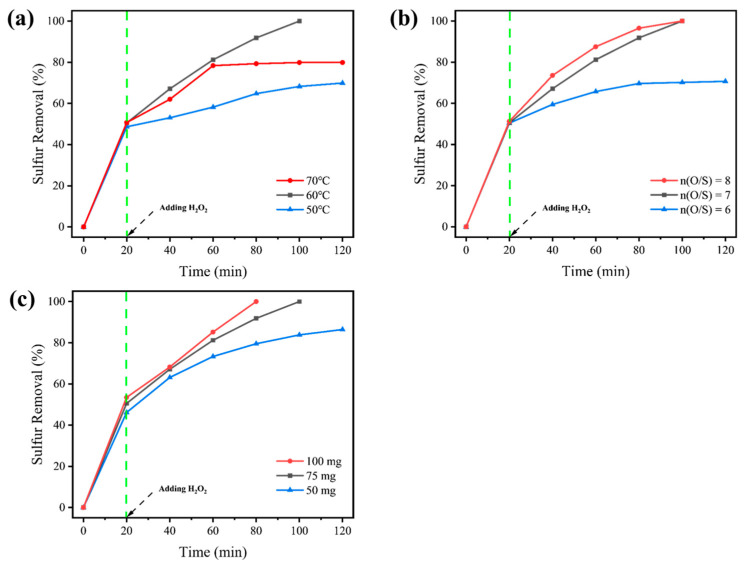
Sulfur removal under different (**a**) reaction temperatures, (**b**) O/S molar ratios, and (**c**) catalyst dosages.

To begin with, sulfur removal at different reaction temperatures was investigated (Figure 6a). The sulfur removal at 20 min was mainly attributed to the extraction ability of MeCN, which was slightly affected by temperature. However, after adding H_2_O_2_, a noticeable difference in sulfur removal at different temperatures could be observed. For sulfur removal conducted at 50 °C, sulfur removal of only 69.9% was received after reaction for 100 min, indicating that a higher reaction temperature is needed. Generally, higher reaction activity will be acquired under higher reaction temperatures. However, for the reaction conducted under 70 °C, no obvious increase in sulfur removal was witnessed after the reaction for more than 80 min. This might be caused by the rapid decomposition of H_2_O_2_ under the higher reaction temperature. Therefore, a reaction temperature of 60 °C would be the optimal setting for this oxidative desulfurization system. Here, deep desulfurization (99.8%, <1 ppm) could be achieved after reaction for 100 min.

As an oxidant used in the oxidative desulfurization process, a higher dosage of H_2_O_2_ can facilitate deep desulfurization within a shorter reaction time. Whereas, achieving deep desulfurization with the lowest H_2_O_2_ consumption is the target we aim for. Here, in this work (Figure 6b), sulfur removal of only 70.7% would be attained after reaction for 100 min with the oxidant/sulfide (O/S) molar ratio of 6. By increasing the O/S molar ratio to 7, deep desulfurization (99.8%) could be achieved after a reaction for 100 min. Although deep desulfurization would also be achieved with an O/S molar ratio of 8, it would not be selected due to economic and safety concerns.

Subsequently, the effect of the catalyst amount added to the reaction system was also investigated (Figure 6c). The sulfur removal at 20 min shows slight differences, which might be attributed to the adsorptive desulfurization capability of the added catalyst, aside from the primary extraction capabilities of the extractant. For the desulfurization using 50 mg catalyst, sulfur removal of only 86.4% could be gained after being reacted for 100 min. For the sulfur removal using 75 mg and 100 mg of catalyst, deep desulfurization could both be achieved. From the consideration of costs, the usage of the catalyst was optimized to 75 mg for further investigations.

### 3.5. Sulfur Removal for Oils with Different Substrates or Sulfur Concentrations

**Figure 7:**
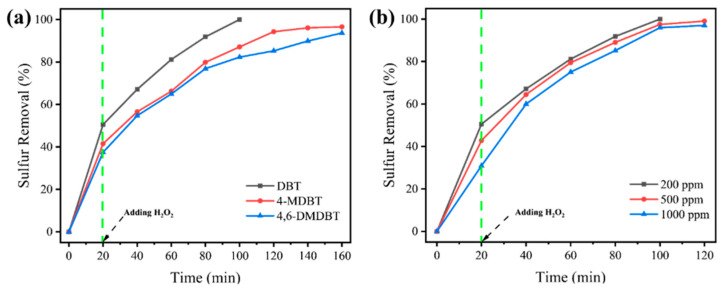
Sulfur removal for oils with (**a**) different substrates and (**b**) different sulfur concentrations.

For the actual unrefined oils, the types and concentrations of sulfide in each oil are different. Thus, another two investigations were conducted subsequently to verify the practicality of this desulfurization procedure. Firstly, sulfur removal for DBT, 4-methyl-dibenzothiophene (4-MDBT), and 4,6-dimethyl-dibenzothiophene (4,6-DMDBT), as the most representative three unreactive sulfides, were explored (Figure 7a). The sulfur removal decreases following the order of DBT > 4-MDBT > 4,6-DMDBT. For 4-MDBT and 4,6-DMDBT, sulfur removal is unsatisfactory after a reaction of 100 min. By further extending the reaction time, sulfur removal for the above two sulfides could reach up to 96.6% and 93.7%, respectively, which would also be a satisfactory level of sulfur removal. Also, the decrease in sulfur removal could be attributed to the increased steric hindrance effect from the additional methyl group and the increased electron cloud density of the S atom [73,74,75].

Subsequently, sulfur removal of oils with higher sulfur concentrations (500 ppm and 1000 ppm) was examined (Figure 7b). The sulfur removal detected around 20 min could be mainly ascribed to the extraction ability of the MeCN extractant. The obvious decrease in sulfur removal at 20 min for oils with higher sulfur concentrations could be attributed to the saturation of DBT in the MeCN extractant. After the introduction of the H_2_O_2_ oxidant, the difference in sulfur removal for different samples decreases significantly, indicating the effective oxidation of DBT to its corresponding sulfone. After a reaction time of 100 min, the sulfur removal decreases slightly with the increase in sulfur concentration, which is superior to our previous work [76]. With an extended reaction time of 20 min, sulfur removal of 99.0% and 97.1% was achieved for oils with sulfur concentrations of 500 ppm and 1000 ppm, respectively. The slight decrease in sulfur removal could be attributed to the increased generation of H_2_O during the reaction process, as more H_2_O_2_ oxidant is needed to maintain the same O/S molar ratio.

The exceptional sulfur removal for oils containing various refractory sulfides or higher sulfur concentrations illustrates the practicality of this oxidative desulfurization system.

### 3.6. The Recycling Performance of the Reaction System

**Figure 8:**
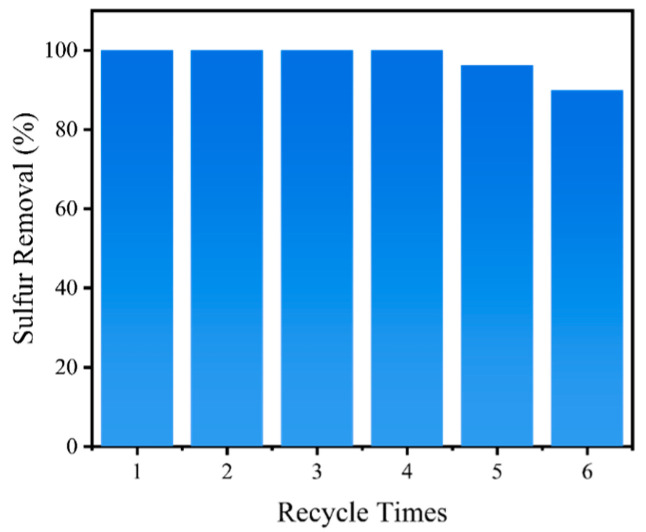
Recycling performance of the designed oxidative desulfurization system.

The recycling performance of the synthesized catalyst is another indicator for assessing its potential for practical application (Figure 8). After each oxidative reaction, the oxidized oil phase was separated. The catalyst was then washed with ethanol, followed by drying in an oven before being used in the next run. Fresh oil and oxidant were then added for the next cycle. Deep desulfurization can still be achieved in the first four cycles. However, sulfur removal decreases to 96.2% in the fifth cycle and drops sharply to 90.0% in the sixth cycle. This may be attributed to the saturation of oxidized sulfones in the extractant phase, which hinders effective contact between the sulfides and the oxidant, eventually reducing sulfur removal. Taken together, this reaction system exhibits a certain degree of cycling stability.

## 4. Conclusions

In summary, UiO-66 based catalysts with defects and nitro functional groups could be synthesized via a facile approach. The structure of the constructed catalyst was characterized using XRD, SEM, FT-IR, and Raman techniques. The measurements indicate that UiO-66 with unsaturated metal sites and -NO_2_ group could be synthesized successfully, and the basic structure of the UiO-66 framework could be retained. The efficient construction of a catalyst with unsaturated metal sites and electron-withdrawing function groups contributes to the enhancement of catalytic oxidation performance. Here, deep desulfurization (99.8%, <1 ppm) could be achieved under relatively mild reaction conditions (60 °C and atmospheric pressure) compared to traditional industrial HDS processes. Additionally, this reaction system also shows desirable sulfur removal for oils with much higher sulfur concentrations and oils with other unreactive sulfides. Given its convenient synthesis approach and outstanding desulfurization performance, the elite catalyst in this work is considered to be a promising candidate for the novel desulfurization process.

## Data Availability

Data are contained within the article and Supplementary Materials.

## Supplementary Materials

The following supporting information can be downloaded at https://www.mdpi.com/article/10.3390/nano15120931/s1, Experimental section. Table S1: BET surface areas (m^2^/g) of the prepared UiO-66-based catalysts; Scheme S1: Illustration of the influence of nitro substituents and unsaturated Zr sites on the desulfurization mechanism; Scheme S2: The reaction of DBT oxidation using pristine UiO-66 and modified D-UiO-66 as catalysts and H_2_O_2_ as an oxidant.
