# Supplementary material for: The Facile Construction of Defect-Engineered and Surface-Modified UiO-66 MOFs for Promising Oxidative Desulfurization Performance
Source: Nanomaterials (Basel). 2025 Jun 15;15(12):931. doi: 10.3390/nano15120931 (PMC12195884; doi:10.3390/nano15120931)
Supplement: Supplementary file 1 [file nanomaterials-15-00931-s001.zip › nanomaterials-3599317-supplementary.pdf]

The facile construction of defect engineered and surface modified UiO-66 MOFs for promising oxidative desulfurization performance

Chao Wang [1,*], Junchao Ding [1], Haoyu Wu [1], Jiaxuan Zhang [1], Jing Xu [1], Ying Zhang [1], Mindan Ma [1], Ming Zhang [2], Hongping Li [2,*]

[1] School of the Environment and Safety Engineering, School of Emergency Management, Jiangsu University, Zhenjiang 212013, P.R. China

[2] Institute for Energy Research, Jiangsu University, Zhenjiang 212013, P.R. China

E-mail: chaowang@ujs.edu.cn (Chao Wang); hongpingli@ujs.edu.cn (Hongping Li)

Experimental section:

Determination of characterization: Powder X-ray diffraction (XRD) patterns were obtained by using an X-ray diffractometer (Shimadzu 6100) equipped with copper and palladium, and scanning was carried out at a speed of 5°/min in the range of 2θ = 5°-50°. The micromorphology of the catalyst was collected by a field emission scanning electron microscope (SEM, JSM-IT800). The Raman spectrum was recorded by a laser microscope Raman spectrometer (DXR). The water contact angles were calculated by droplet shape analyzer (Krüss DSA 100) at room temperature and ambient relative humidity. A Nicolet Nexus 470 Fourier-transform infrared (FT-IR) spectroscopy was used to record the spectra of samples, and the FT-IR spectra were collected from 4000 cm^{-1} to 400 cm^{-1}. The mass changes of the catalyst during heating were determined using a Netzsch TG209F1 Libra thermogravimetric analyzer (TGA). The Brunauer-Emmett-Teller (BET) surface area analysis were performed utilising an ASAP 2460 device from Micromeritics.

Preparation of model oil: DBT (0.878 g) and hexadecane (3.056 g) were added to solvent dodecane. After the solid was dissolved completely, the solution was transferred to a 1000 mL volumetric flask and made up to volume. The prepared solution is the model oil with DBT as sulfide with a sulfur content of 200 ppm. The

model oils with initial sulfur concentrations of 500 ppm and 1000 ppm, using DBT as the substrate, were prepared with 2.195 g and 4.390 g of DBT, respectively. Additionally, model oils using 4-MDBT and 4,6-DMDBT as substrates were prepared with 0.945 g of 4-MDBT and 0.950 g of 4,6-DMDBT, respectively.

The detection of sulfur concertation: The sulfur concentration of oil at time t was detected using a gas chromatography (GC) instrument. The temperature of the GC process started at 100°C and increased to 200°C at 15°C/min. Injector temperature was 300°C and detector temperature was 250°C. The temperature program of the GC-MS process started at 100°C and rose to 200°C at 15°C/min. The injector temperature was 250°C.

Table S1. BET surface areas (m^2/g) of the prepared UiO-66-based catalysts.

Catalysts	UiO-66	D-UiO-66	UiO-66-NO_2	D-UiO-66-NO_2
BET surface areas (m^2/g)	972	1168	1053	820

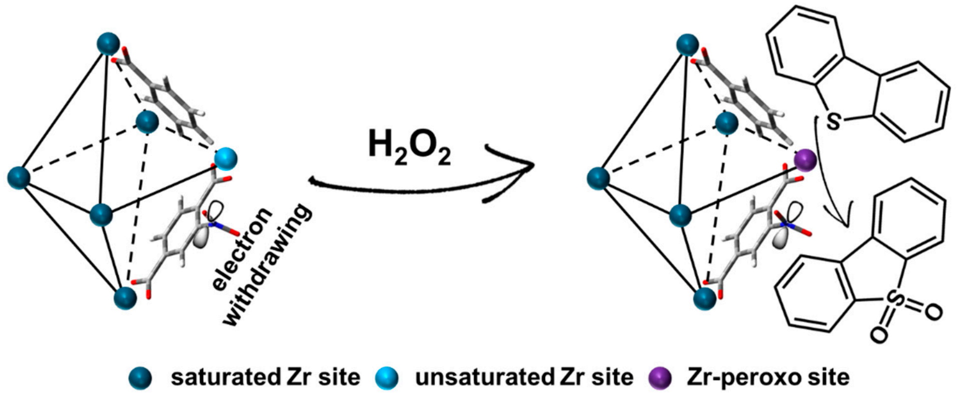

Scheme S1. Illustration of the influence of nitro substituents and unsaturated Zr sites on the desulfurization mechanism.

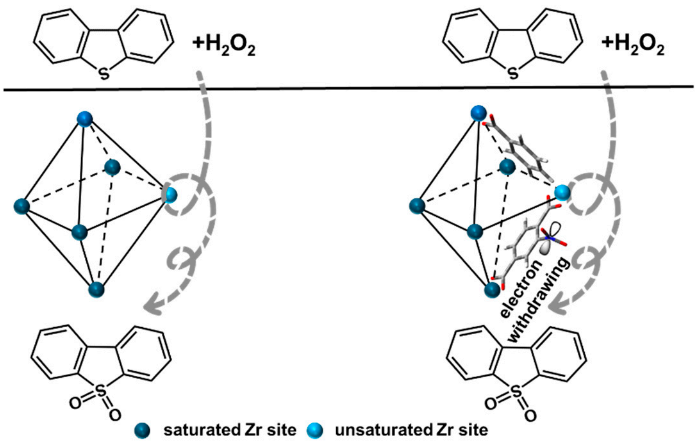

Scheme S2. The reaction of DBT oxidation using pristine UiO-66 and modified D-UiO-66 as catalysts and H_2O_2 as an oxidant.
